# Skin infection, housing and social circumstances in children living in remote Indigenous communities: testing conceptual and methodological approaches

**DOI:** 10.1186/1471-2458-5-128

**Published:** 2005-12-08

**Authors:** Ross S Bailie, Matthew R Stevens, Elizabeth McDonald, Stephen Halpin, David Brewster, Gary Robinson, Steven Guthridge

**Affiliations:** 1 Menzies School of Health Research and Institute of Advanced Studies, Charles Darwin University, Darwin, Australia; 2 Flinders University Northern Territory Clinical School, Darwin, Australia; 3 School for Social and Policy Research, Institute of Advanced Studies, Charles Darwin University, Darwin, Australia; 4 Northern Territory Department of Health and Community Services, Darwin, Australia

## Abstract

**Background:**

Poor housing conditions in remote Indigenous communities in Australia are a major underlying factor in poor child health, including high rates of skin infections. The aim of this study is to test approaches to data collection, analysis and feedback for a follow-up study of the impact of housing conditions on child health.

**Methods:**

Participation was negotiated in three communities with community councils and individual participants. Data were collected by survey of dwelling condition, interviews, and audit health centre records of children aged under seven years. Community feedback comprised immediate report of items requiring urgent repair followed by a summary descriptive report. Multivariate models were developed to calculate adjusted incidence rate ratios (IRR) for skin infections and their association with aspects of household infrastructure.

**Results:**

There was a high level of participation in all communities. Health centre records were inadequate for audit in one community. The records of 138 children were available for development of multivariate analytic models. Rates of skin infection in dwellings that lacked functioning facilities for removing faeces or which had concrete floors may be up to twice as high as for other dwellings, and the latter association appears to be exacerbated by crowding. Younger children living in older dwellings may also be at approximately two-fold higher risk. A number of socioeconomic and socio-demographic variables also appear to be directly associated with high rates of skin infections.

**Conclusion:**

The methods used in the pilot study were generally feasible, and the analytic approach provides meaningful results. The study provides some evidence that new and modern housing is contributing to a reduction in skin infections in Aboriginal children in remote communities, particularly when this housing leads to a reduction in crowding and the effective removal of human waste.

## Background

Bacterial skin infections are a common and important cause of morbidity in disadvantaged populations. In Indigenous communities in the Northern Territory the prevalence has been reported at between 10 and 70% [1-4]. Pyoderma is important not only because of its local effects as a skin infection, but more importantly because the primary pathogen underlying skin infection in Aboriginal children is a Group A Streptococcus (GAS) [5]. GAS infections of the skin are believed to be an important factor in acute post-streptococcal glomerulonephritis (APSGN) and acute rheumatic fever (ARF) [2,4,6,7]. Rates of ARF and consequent rheumatic heart disease (RHD) in Aboriginal children living in these communities are reported to be among the highest in the world [8-10]. GAS is also believed to contribute to the high rates of chronic renal failure in these communities [11]. Pyoderma is believed to be the major source of invasive GAS disease. Scabies infestation is believed to underlie between 50–70% of cases of pyoderma [1], and has a reported prevalence among children of around 50% [1,12].

The underlying determinants of these high rates of pyoderma are reported to include crowding [13,16], inadequate water supply [17,18], heat and humidity [19,20], poor education and poor hygiene [19,21-23]. The interdependence of these and other socio-economic factors has led to difficulty in assessing the relative importance of such factors [13,17]. This difficulty applies to diverse aspects of health in these communities and not only to skin health in children.

**Figure 1:**
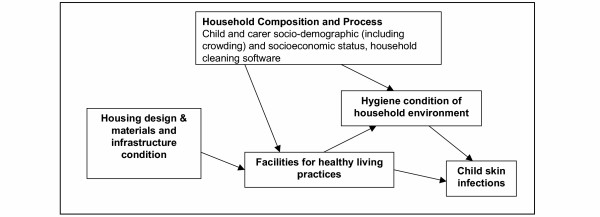
Conceptual Framework relating household composition and processes, infrastructure condition, hygiene condition and childhood skin infections.

An important aspect of the environment of these children that is potentially amenable to relatively immediate intervention is the quantity and quality of housing. A major objective of housing programs in Indigenous communities is the improvement of health. Work on defining components of household infrastructure important to the conduct of a set of 'Healthy Living Practices' (HLPs) [24,25] has been influential in the development of housing programs in remote communities. Components of household infrastructure relevant to the HLPs, including those important to the prevention of skin infections, are in poor condition in many remote Indigenous Australian communities [26-28]. However, there is a lack of empirical data that can be used to inform how housing design can achieve the most significant gains in health, and to understand other factors that may moderate or mediate potential health improvements. Understanding these other factors, and developing programs to address them, should contribute to ensuring housing improvements flow through to improved health. A simple conceptual representation categorises these factors as (1) infrastructure, (2) household composition and social process and (3) condition of household environment (Figure 1).

This paper reports on the findings of a pilot study conducted in the lead up to a before and after controlled study of the impact of provision of better quality housing on the health of children in eleven remote Aboriginal communities. The objectives of the pilot study were to test data collection procedures, to refine specification of variables, to test the conceptual framework for the study and to refine the approach to the analysis and community feedback procedures (community feedback is not discussed in this paper). The objectives of the analysis were to examine the association between skin infections in Aboriginal children living in remote communities and the functional status of basic items of household infrastructure, and to examine the extent to which these associations are mediated by other household and carer characteristics.

## Methods

### Study setting and agreement to participate

Three remote communities were approached to participate in the study. Communities were selected to ensure some variation in size, development and geographic spread. The three communities typified the very poor environmental conditions prevalent in remote Indigenous communities in Australia. Participation was initially negotiated with community councils, and individual consent was subsequently provided by individual participants. Two communities were located in the Top End of the Northern Territory and the third was located in Central Australia. The study was approved by the Top End and Central Australian Human Research Ethics Committees and by the associated Indigenous health research sub-committees.

### Survey Processes

Three data collection processes were used: 1) a survey of dwelling condition; 2) interviews with the main householder of each dwelling and with the main carer of each child aged under seven years; and 3) an audit of health centre records. All dwellings in each community were included in the housing survey. Data on dwelling condition and the availability of cleaning materials were obtained through an inspection of the dwelling by an environmental health officer or housing officer. The functional state of each item was scored on a previously described five point ordinal scale using standardised survey forms and protocols developed for housing program management purposes and refined through evaluations of the survey process and survey data for three consecutive annual surveys [26,29]. Over 90 infrastructure items in and around the dwelling were examined in the survey, and where appropriate, physically tested. Items that were present and required no more than minor repairs were defined as 'functional'. Information on items requiring immediate or urgent repair were reported to the community housing office at the time of the survey and a report on the general state of community housing was provided following more detailed analysis of the survey data.

Children were eligible for inclusion in the study if they were less than seven years of age and had spent at least six of the previous 12 months living in the community. For all dwellings with at least one eligible child data on socioeconomic and demographic variables were collected through structured face-to-face interviews with the main householder of each dwelling and with the main carer of each eligible child. Where the main householder or carer was not available the interview was conducted with a secondary householder or carer. The definition of study variables is included in Table 1. Research personnel included a research officer with wide experience as a remote area nurse and health service manager with the assistance of an Aboriginal man with experience in Aboriginal community housing management. They were accompanied and assisted by a community resident employed for this purpose through the community council.

**Table 1:** Definition and categories of variables

**Variable**	**Description and categories**
***Outcome variable***	
Skin infection incidence rate (person-year)	For each child aged under seven years, data on presentations to the health centre for skin infections (scabies and/or bacterial infection) were collected through an audit of health centre records for the one year period preceding the survey. Any record in the clinical notes indicating a diagnosis of scabies, impetigo or infected skin sores was counted as an episode, except if the record was within seven days of an earlier diagnosis of the same condition.
***Primary explanatory variables***	
*Housing condition, materials and design*	
Facilities for washing children not functioning	1. For *child <1 year*: (i) bathroom basin, hot tap, cold tap, bench, door, electrical and general structure are all functioning, or (ii) kitchen sink, hot tap and cold tap all functioning. 2. For *child aged 1 to <3 years*: (i) laundry trough, hot tap, cold tap, shelf, electricity, floor drainage and general structure are all functioning, or (ii) the bathroom shower head, hot tap, cold tap, drainage, bench, electrical and general structure are all functioning. 3. For *child aged 3 to less than 7 years*: bathroom shower head, hot tap, cold tap, drainage, bench, door, electrical and general structure are all functioning. Facilities not functioning versus facilities functioning (reference).
Facilities for washing clothes and bedding not functioning	1. Laundry trough, hot tap, cold tap, shelf, electricity, floor drainage and general structure all functioning. Facilities not functioning versus facilities functioning (reference).
Facilities for removing human faeces not functioning	1. If *child <1 year*: toilet pan, cistern, water supply, drainage, bathroom basin and hot and cold taps are all functioning. 2. If *child aged 1 to <3 years*: child toilet equipment (e.g. potty – small plastic toilet), toilet pan, cistern, water supply, drainage, electricity, general structure, bathroom basin and hot and cold taps are all functioning. 3. If *child aged 3 to less than 7 years*: toilet door, electricity, general structure, toilet pan, cistern, water supply, drainage, bathroom basin, hot tap and cold tap are all functioning. Facilities not functioning versus facilities functioning (reference).
Combined HLPs	Failed at least one of the three healthy living practices previously defined
Concrete/other floor material (no tiles)	Observed by surveyor. The floor type for the main living areas (kitchen and living room) of dwellings were categorised according to (1) concrete, (2) tiles, (3) other, (4)concrete and tiles, (5) concrete and other, and (6) tiles and other. The variable was then dichotomised to reflect the contrast between dwellings that have concrete/other flooring versus those that have tiles (reference).
Dwelling built pre 1980	As reported by community housing office or experienced government housing program staff. Older dwellings (pre 1980) versus newer dwellings (reference).
***Secondary explanatory variables***	
Community A	Community A versus Community B (reference).
*Hygiene condition of dwelling environment and immediate surrounds*	
Internal contaminants present	Presence of obvious organic contaminants observed by surveyor in the dwelling (e.g. faecal contamination (disposable nappies), food scraps, etc.) versus no contaminants (reference).
External contaminants present	Presence of obvious organic contaminants observed by surveyor on the sealed surrounds (i.e. veranda) of the dwelling versus no contaminants (reference).
*Household cleaning software*	
Household cleaning equipment missing	Observed by surveyor. At least one of the broom, mop or bucket absent (as observed by the surveyor) versus dwellings with all items present (reference).
No soap in dwelling	Observed by surveyor. No soap around the bathroom, kitchen or laundry sinks versus soap present (reference).
*Child and carer socio-demographic factors*	
Child age less than 3 years	Reported by carer and date of birth verified against health centre audit (health centre records used as true age). Child less than 3 years versus child 3 to less than 7 years (reference).
Child sex (male)	Reported by carer. Child sex male versus female (reference).
Carer age	Reported by carer. Categorised into three dummy variables: carer less than 20 years, carer 20 to 34 years (reference), and carer 35 plus years.
Low family income	Family income per week as reported by the primary carer. Low family income (less than median family income ($935) for the two communities) versus greater than median family income (reference).
Carer highest education	Highest formal qualification as reported by the carer. Categorised to three dummy variables. Grades 11 and 12 (reference), grades 9 and 10, and grade 8 and below.
Carer unemployed	Reported by carer. Employed refers to any employment including CDEP (a type of 'work-for-the-dole' scheme). Carer unemployed versus carer employed (reference).
*Household composition*	
Four or more children aged less than 7 years in the dwelling	Number of children less than 7 years usually resident in the dwelling (at least 6 out of 12 months) as reported by householder and primary carer. Four or more children less than 7 years in dwelling versus less than 4 children (reference).
Three carers' in dwelling	Reported by householder and carer. Only the main carer of each child was counted (i.e. variable refers to number of carers of different children). Three carers in dwelling versus one or two carers' in dwelling (reference).
Crowding: Residents per bedroom	Number of residents (includes visitors) reported by householder and carer. Number of bedrooms based on dwelling survey. Categorised into three dummy variables. Two persons or less per bedroom (reference), greater than two to four or less persons per bedroom, and greater than 4 persons per bedroom.
Child mobility (high)	Reported by carer whether child sleeps at another dwelling at least one night in ten (or 10%). Child mobility high versus low mobility (reference).
Presence of a visitor in the dwelling	Reported by householder and carer. A visitor was defined as anyone who is not a usual resident who is currently staying at the dwelling for at least one week. Visitor(s) present versus no visitors (reference).

For each child, data on presentations to the health centre for skin infections and other specific conditions were collected through an audit of health centre records for the one year period preceding the survey. In community C the recording of presentations in health centre records of children with common childhood conditions was inadequate for audit purposes. This community was therefore excluded from analysis of the occurrence of skin infection.

### Data analysis

Our analysis of the relationship between child skin infections and household composition and process, condition of household environment, and dwelling infrastructure follows the conceptual model presented in Figure 1. Composite variables reflecting the functionality of the infrastructure required for each of the three healthy living practices [24-26,28] that were hypothesised to be of primary importance to skin infections (wash children, wash clothes, removal of faeces) and a combined HLP variable were constructed (Table 1). Decisions regarding the infrastructure required for each healthy living practice for children of different ages were based on discussions with Indigenous and non-Indigenous people with wide experience of maternal and child health and housing programs in remote Indigenous communities.

Counts and percentages were calculated for all explanatory variables by community and differences were assessed using Fisher's exact test. Skin infection incidence rates (person-year) were tested for over dispersion to determine whether Poisson or negative binomial regression was used to model the counts of presentations with a skin infection diagnosis. All regression models were adjusted for clustering of children within dwellings using the Huber-White sandwich variance estimator [30]. Univariate incidence rate ratios were calculated for all explanatory variables.

The multivariate analysis examines how different aspects of the housing in which children lived related to rates of skin infection while controlling for the effects of other household composition and processes (see figure 1 and table 1). Dwelling infrastructure variables included the three HLP variables, the combined HLP variable, dwelling age (built pre/post 1980), and the floor type (tiles or concrete/other). A main effects multivariate regression model using stepwise backward elimination with a probability of 0.1 for exclusion of variables was developed. After main effects models were established, clinically or conceptually justifiable interaction terms (first order effects) were entered starting with the most significant interaction term. Only interactions where the cross tabulation had at least 3 observations per cell were tested to avoid spurious incidence rate ratios or a lack of convergence in the iterative regression estimation process. If the term added to the explanatory power of the model, it was included in the final model. When final models were determined, contrasts for each interaction term(s) were tested to assess those that were significantly associated with skin infection incidence rates.

Four models were developed (Table 4) to tease out significant associations of different aspects of household infrastructure with the incidence of skin infections, the factors that mediate or moderate these associations and identify other factors that are directly associated with skin infections. Model 1 included all the explanatory variables from table 1 (excluding combined HLP due to multi-collinearity). Model 2 used the combined HLP variable instead of the three separate HLP variables. Model 3 substitutes the dwelling age and flooring material variables for the HLP variables. Model 4 includes the three separate HLP variables and no other infrastructure variables. All statistical analyses were carried out using Stata version 8.2.

## Results

Complete housing survey and interview data were obtained for 161 of an estimated 212 (76%) children in the eligible age range from the three communities. For the remaining estimated 51 children, the household residents declined to participate, were known to be away for an extended period or were not available for interview on at least three repeat visits. The analysis of skin infection data included the records of 138 children living with 80 carers in 69 dwellings in two communities. Levels of crowding and the functional state of household infrastructure varied between communities (Table 2).

**Table 2:** Summary statistics for each study community: Primary explanatory variables (at the dwelling level), and population and housing variables

	**Community A**	**Community B**	**Community C**	**Total**
	
*Population and housing*				
Estimated number of children aged < 7 years	135	54	23	212
Number of children < 7 years surveyed	100	38	23	161
Estimated response rate	*74.1*	*70.4*	*100.0*	*75.9*
Number of carers^§^	54	26	15	95
Number of residents^¶^	473	175	102	750
Number of usual residents	420	157	87	664
Number of dwellings	46	23	13	82
Number of bedrooms	132	58	36	226
Mean no. of bedrooms per dwelling (SD)	2.9 (0.5)	2.5 (0.8)	2.8 (0.8)	2.8 (0.7)
Mean no. of residents per bedroom (SD)	3.6 (1.5)	3.4 (1.6)	2.7 (1.4)	3.4 (1.5)
*Primary explanatory variables*				
*Number of dwellings (%)*	***n (%)***	***n (%)***	***n (%)***	***n (%)***
	
Facilities not functioning to:				
Wash children	15 (32.6)	1 (4.3)	7 (53.8)	23 (28.0)
Wash clothes	21 (45.7)	5 (21.7)	5 (38.5)	31 (37.8)
Remove faeces	21 (45.7)	7 (30.4)	12 (92.3)	40 (48.8)
Combined healthy living practices	29 (63.0)	10 (43.5)	12 (92.3)	51 (62.2)
Concrete/other floor material	19 (41.3)	1 (4.3)	11 (91.7)	31 (37.8)
Dwelling built pre 1980	8 (17.4)	8 (34.8)	5 (38.5)	21 (25.6)

§ Carers' of children less than 7 years

¶ Residents refers to usual residents plus visitors (see Table 1).

Almost 40% (54) of children had no health centre presentations for skin infection over the study period, while over a third (47) had two or more presentations and 10% (14) had five or more (Table 3). Explanatory factors showing considerable variation between the two communities included the availability of household cleaning equipment, availability of soap, family income, carers' employment status, carers' education and crowding.

**Table 3:** Summary statistics for explanatory variables and univariate incidence rate ratios (IRRs) for presentation with skin infections: Confidence intervals adjusted for clustering of children in dwellings.

	**Community A (N_A _= 100)**	**Community B (N_B _= 38)**	**All children (N = 138)**	**Univariate incidence rate ratios**
	
	**n (%)**	**n (%)**	**n (%)**	**IRR (95% CI)**
No skin infections	41 (41.0)	13 (34.1)	54 (39.1)	na
2 or more skin infections	37 (37.0)	10 (26.3)	47 (34.0)	na
5 or more skin infections	12 (12.0)	2 (5.3)	14 (10.0)	na
Total number of skin infections	162	50	212	na
Median skin infections per child (range)	1 (0 – 8)	1 (0 – 9)	1 (0 – 9)	na
Skin incidence rate (person-years)	1.84	1.47	1.74	na
***Primary explanatory variables ***				
Dwelling facilities required to:				
- wash children not functioning	35 (35.0)	3 (7.9) **	38 (27.5)	1.23 (0.75 – 1.99)
- wash clothes not functioning	51 (51.0)	10 (26.3) *	61 (44.2)	1.40 (0.83 – 2.36)
- remove human faeces not functioning	42 (42.0)	8 (21.1) *	50 (36.2)	1.68 (0.99 – 2.87)
Combined HLPs	64 (64.0)	15 (39.5) *	79 (57.2)	**1.96 **(1.25 – 3.07)
Concrete/other floor material (no tiles)	53 (53.0)	2 (5.3) **	98 (71.0)	1.26 (0.74 – 2.14)
Dwelling built pre 1980	15 (15.0)	14 (36.8) **	29 (21.0)	1.23 (0.68 – 2.25)
***Secondary explanatory variables***				
Community A	-	-	100 (72.5)	1.27 (0.74 – 2.20)
Community B (reference category)	-	-	38 (27.5)	1.00
*Health software*				
Household cleaning equipment missing	79 (79.0)	6 (15.8) **	87 (63.0)	1.59 (0.90 – 2.80)
No soap in bathroom	57 (57.0)	5 (13.2) **	62 (44.9)	1.23 (0.72 – 2.09)
*Contaminants*				
Internal contaminants present	79 (79.0)	35 (92.1)	114 (82.6)	0.57 (0.28 – 1.14)
External contaminants present	82 (82.0)	36 (94.7)	118 (85.5)	1.66 (0.83 – 3.31)
*Child and carer socio-demographic factors*				
Child less than 3 years	49 (49.0)	19 (50.0)	68 (49.3)	**1.95 **(1.40 – 2.72)
Child sex (male)	52 (52.0)	17 (44.7)	69 (50.0)	0.86 (0.52 – 1.43)
Child mobility (high)	7 (7.0)	5 (13.2)	12 (8.7)	0.78 (0.42 – 1.47)
Carer age < 20 years	13 (13.0)	3 (7.9)	16 (11.6)	**3.20 **(2.18 – 4.71)
Carer 20 years to LT 35 years	77 (77.0)	29 (76.3)	106 (76.8)	1.00
Carer 35 years or more	10 (10.0)	6 (15.8)	16 (11.6)	**1.79 **(1.06 – 3.04)
Low family income (≤ $935 or median)	58 (58.0)	12 (31.6) **	70 (51.1)	**2.10 **(1.30 – 3.40)
Carer unemployed	76 (76.0)	9 (23.7) **	85 (61.6)	1.25 (0.78 – 1.99)
Carer highest level of education				
Years 11 or 12	11 (11.0)	6 (15.8)	17 (12.3)	1.00
Years 9 or 10	23 (23.0)	21 (55.3) **	44 (31.9)	1.26 (0.74 – 2.16)
Year 8 or below	66 (66.0)	11 (29.0) **	77 (55.8)	**1.92 **(1.19 – 3.12)
*Household composition variables*				
Four or more children < 7 yrs in dwelling	29 (29.0)	8 (21.1)	37 (26.8)	1.54 (0.84 – 2.82)
Three carers in dwelling	12 (12.0)	4 (10.5)	16 (11.6)	**2.26 **(1.24 – 4.13)
Visitors present in dwelling	20 (20.0)	12 (31.6)	31 (22.6)	0.79 (0.49 – 1.28)
Residents per bedroom				
Lowest (1.33–2.00)	9 (9.0)	10 (26.3) *	19 (13.8)	1.00
Middle (2.33–4.00)	57 (57.0)	16 (42.1)	73 (52.9)	1.51 (0.59 – 3.86)
Highest (4.33–8.33)	34 (34.0)	12 (31.6)	46 (33.3)	1.11 (0.44 – 2.79)

Difference between two communities: * = p ≤ 0.05; ** = p ≤ 0.01.

**Table 4:** Multivariate adjusted incidence rate ratios for skin infections using a negative binomial model adjusted for clustering of children in dwellings.

	**Model 1**	**Model 2**	**Model 3**	**Model 4**
	
	**IRR (95% CI)**	**IRR (95% CI)**	**IRR (95% CI)**	**IRR (95% CI)**
*Main effects*				
Facilities for removing human faeces not functioning	1.28 (0.88–1.85)	na	na	2.03 (1.34–3.06)
Combined HLPs	na	1.37 (0.92–2.03)	na	na
Dwelling built pre 1980^1,2,3^	0.76 (0.36–1.61)	0.64 (0.32–1.30)	1.16 (0.51–2.60)	na
Concrete/other floor material^1^	2.27 (0.67–7.67)	a	a	na

Household cleaning equipment missing^3^	a	a	1.82 (1.18–2.81)	a
Internal contaminants present	a	a	a	0.67 (0.45–1.00)
External contaminants present	a	a	a	2.27 (1.28–4.03)
Child age				
Less than 3 years^1,2,3^	1.57 (1.06–2.33)	1.46 (0.98–2.18)	1.54 (1.04–2.29)	1.63 (1.17–2.28)
3 to less than 7 years	1.00	1.00	1.00	1.00
Carer age				
Less than 20 years	2.29 (1.60–3.26)	2.36 (1.54–3.63)	2.19 (1.45–3.29)	a
20 to 34 years	1.00	1.00	1.00	a
35 years or more	2.41 (1.57–3.71)	2.30 (1.56–3.39)	2.38 (1.64–3.44)	a
Three carers in dwelling^4^	a	a	a	0.63 (0.21–1.90)
4 or more children under 7 years	2.55 (1.70–3.81)	1.95 (1.26–3.02)	1.89 (1.23–2.92)	a
Child mobile	1.39 (0.88–2.20)	1.46 (0.90–2.36)	1.57 (0.98–2.50)	a
Residence per bedroom				
Lowest (≤ 2 per bedroom)	1.00	1.00	1.00	a
Middle (2 to ≤ 4 per bedroom)^1^	2.22 (0.89–5.52)	1.35 (0.72–2.52)	1.25 (0.70–2.26)	a
Highest (4 to 8.33 per bedroom)^1^	1.61 (0.61–4.21)	1.81 (0.90–3.63)	1.54 (0.81–2.93)	a
Carers education				
Grade 11 and 12	a	a	a	1.00
Grade 9 and 10	a	a	a	0.92 (0.53–1.61)
Grade 8 and below	a	a	a	1.70 (1.02–2.84)
Low family income (≤ $935)	2.21 (1.53–3.20)	1.84 (1.25–2.69)	1.78 (1.24–2.57)	1.62 (1.10–2.37)
Carer unemployed^4^	0.73 (0.52–1.03)	a	a	0.53 (0.35–0.82)

*Interactions*				
Child less than 3 years × dwelling built pre-1980	3.04 (1.52–6.08)	3.63 (1.74–7.57)	4.14 (1.95–8.80)	na
Cleaning equipment missing × dwelling built pre-1980	a	a	0.36 (0.17–0.74)	na
Concrete/other floor material × middle level crowding	0.35 (0.09–1.30)	a	a	na
Concrete/other floor material × highest level crowding	1.11 (0.29–4.26)	a	a	na
Carer unemployed × three or more carer's in dwelling	a	a	a	6.19 (1.93–19.84)

Log pseudo-likelihood	-196.298	-201.777	-199.553	-203.606

Model: Pseudo *R*^2^	15.1%	12.7%	12.7%	11.9%

1 Significant interaction term in model 1

2 Significant interaction term in model 2

3 Significant interaction term in model 3

4 Significant interaction term in model 4

a Variable dropped during backward stepwise elimination (p > 0.10) or non-significant interaction term na Variable not included in backward stepwise elimination process

In the univariate analysis all of the HLP variables showed a positive association with presentations for skin infections in study children, although only *combined HLP *reached statistical significance (Table 3). Other explanatory variables showing significant positive univariate associations with skin infection incidence rates include child less than 3 years, younger carers and older carers, low family income, carers education year 8 or below and having 3 carers in the dwelling.

The multivariate analysis suggests the variables with the strongest and most consistent association with incidence of skin infections are those reflecting household composition and social process. These include carer's age less than 20 or over 35, four or more children under the age of seven in the dwelling, and low family income (Table 4). Each of these variables was significant in at least three of the four models. High child mobility also tended to be associated with an increased risk of skin infection. All models contained significant interaction terms and contrasts for these are presented in Table 5.

**Table 5:** Significant contrasts for interaction terms in models 1 to 4: Incidence rate ratios (95% confidence intervals).

**Model/Significant contrasts for interaction terms**	**Incidence rate ratio (95% CI)**
*Model 1*	
Young children (<3 years) & old dwelling (pre-1980) versus young children & newer dwellings	2.30 (1.57 – 3.38)
Young children (<3 years) & old dwelling (pre-1980) versus old children & newer dwellings	3.62 (2.32 – 5.64)
Young children (<3 years) & old dwelling (pre-1980) versus old children & old dwellings	4.79 (2.59 – 8.86)
Young children (<3 years) & newer dwelling (post-1980) versus old children & newer dwellings	1.57 (1.06 – 2.33)
Young children (<3 years) & newer dwelling (post-1980) versus old children & old dwellings	2.08 (0.98 – 4.42)
High level of crowding & concrete/other floor versus high crowding & tiled floor	2.52 (1.38 – 4.59)
High level of crowding & concrete/other floor versus middle crowding & tiled floor	11.56 (1.50 – 89.3)
High level of crowding & concrete/other floor versus low crowding & tiled floor	4.05 (1.50 – 10.9)
*Model 2*	
Young children (<3 years) & old dwelling (pre-1980) versus young children & newer dwellings	2.33 (1.54 – 3.53)
Young children (<3 years) & old dwelling (pre-1980) versus old children & newer dwellings	3.41 (2.24 – 5.21)
Young children (<3 years) & old dwelling (pre-1980) versus old children & old dwellings	5.30 (2.83 – 9.94)
Young children (<3 years) & newer dwelling (post-1980) versus old children & newer dwellings	1.46 (0.98 – 2.18)
Young children (<3 years) & newer dwelling (post-1980) versus old children & old dwellings	2.27 (1.12 – 4.61)
*Model 3*	
Young children (<3 years) & old dwelling (pre-1980) versus young children & newer dwellings	4.79 (2.48 – 9.28)
Young children (<3 years) & old dwelling (pre-1980) versus old children & newer dwellings	7.40 (3.52 – 15.6)
Young children (<3 years) & old dwelling (pre-1980) versus old children & old dwellings	6.39 (3.30 – 12.4)
Young children (<3 years) & newer dwelling (post-1980) versus old children & newer dwellings	1.54 (1.04 – 2.29)
Cleaning equip^§ ^missing & newer dwelling (post-1980) versus equipment missing & old dwelling	2.43 (1.16 – 5.10)
Cleaning equip^§ ^missing & newer dwelling (post-1980) versus equip^§ ^not missing & newer dwelling	1.82 (1.18 – 2.81)
*Model 4*	
Carer unemployed & 3 carers in dwelling versus carer unemployed & 1 or 2 carers in dwelling	3.90 (2.45 – 6.21)
Carer unemployed & 3 carers in dwelling versus carer employed & 1 or 2 carers in dwelling	2.09 (1.31 – 3.32)
Carer unemployed & 3 carers in dwelling versus carer employed & 3 carers in dwelling	3.31 (1.07 – 10.2)
Carer unemployed & 1 or 2 carers in dwelling versus carer employed & 1 or 2 carers in dwelling	0.54 (0.35 – 0.82)

§ equipment

The infrastructure variables that remain in model 1 include the presence of facilities to remove faeces, the age of the dwelling and the flooring material (Table 4). The association with dwelling age is modified by child age, with young children living in older dwellings appearing to be at high risk (Table 5). The association with flooring material is modified by crowding, with children living in dwellings with high levels of crowding and un-tiled floors apparently at high risk (Table 5).

Of the three HLP variables, having the facilities to remove faeces appears most important (Table 4). In model 2, where the separate HLP variables are excluded from the model to allow for inclusion of *combined HLP*, the combined variable was forced to remain in the model, but did not reach statistical significance.

Model 3 is similar to model 2 with the exception that *missing household cleaning equipment *replaces *combined HLP*. As with models 1 and 2, the association of this variable with the incidence of skin infections in children was modified by the age of the dwelling, and the association with missing cleaning equipment was stronger for children living in newer, rather than older dwellings (Table 5).

In model 4 where *flooring material *is excluded, the HLP variable reflecting facilities to remove faeces shows a statistically significant association with the incidence of skin infections. This model also indicates the potential importance of a number of other variables that dropped out of the first three models. The presence of organic contaminants in the immediate dwelling surrounds shows a direct association with skin infections, as does lower educational status of the carer (Table 4). This model also indicates that carer's employment status is modified by the number of carers in the dwelling. Children of unemployed carers in dwellings with three or more carers appear at high risk of skin infections (Table 5). Carers' education level also remained in this model, replacing carers' age from models 1 to 3.

## Discussion

The study demonstrated high levels of willingness to participate by community residents, with the main reason for non-participation being extended absence from the community. Very few residents declined to participate in housing surveys or interviews. The quality of child health records in two of the three communities proved adequate for the purposes of the study. The analysis of the data from the two communities for which skin infection data were available indicates that a number of the housing infrastructure variables defined and measured in this study are associated with the occurrence of skin infections in children. The positive independent association between some measures of quality of household infrastructure and skin infections in children is consistent with the general understanding of the importance of housing to health [31-34]. The multivariate analysis provides some insight into the pathways whereby housing conditions may increase risk of skin infections in the remote Indigenous community environment, and is generally supportive of the simple conceptual framework used in the study.

The infrastructure variables most strongly (IRR approaching or more than 2) and consistently (the same or a related variable retained in more than one of the four models) associated with skin infections were the functional state of facilities to remove faeces and the age of the dwelling, although the association with dwelling age was strongly modified by child age. Type of flooring appears to have an important independent association with the occurrence of skin infections. This is evident in the large IRR for contrasts for interaction terms, (Table 5) particularly in crowded dwellings. While older dwellings (built pre 1980) appear to pose a higher risk, it is not clear from this study what characteristics of older dwellings are responsible for this increased risk. The HLP variables that reflect the functional status of facilities for washing clothes and washing children both tended to be associated with higher rates of skin infection in the univariate analysis, but neither of these variables remained in any of the multivariate models.

The variables categorised under the general heading of household composition and social process tended to show the strongest and most consistent associations with increased risk of skin infections. Of particular note and consistent with other studies of child health are the risks posed by crowding of young children and their carers, younger carers *and *older carers, higher child mobility, lower educational attainment, and lower family income. While this supports the importance of a number of socio-economic variables that have direct associations with the occurrence of skin infections and which may modify the effect of housing infrastructure [35,36], it also suggests more specific insights into the social conditions that may contribute to increased health risks for children in these communities.

Because of the cross sectional nature of the data, this study, as with much housing research internationally, has limited capacity to infer causation [31,37-39]. Other limitations relate to sample size and definition and measurement of variables. A more detailed analysis of risk for specific age groups of children could be important because the risks posed by different aspects of household infrastructure may vary according to child age and developmental stage. Our sample size limited the extent to which this was possible. The sample size also limited the potential to explore the extent to which the risk factors for scabies and bacterial skin infections may differ, or the extent to which the findings are driven by a small number of individuals with multiple infections. However, this latter situation is unlikely as almost 60% of children had at least one skin infection and only 14 (10%) had five or more. The inadequacy of the health records in one of the three communities reduced the potential sample size, although this community was the smallest of the three and the reduction in sample size was therefore relatively minor. The generalisability of the study findings may also be limited by the focus on two selected communities. However, the significant differences between communities across a range of explanatory variables (Tables 2 and 3) indicate these factors may be amenable to intervention.

The multivariate models utilise a large number of variables, including interaction terms, on a relatively small dataset. As such, caution is advised when interpreting these results as the model may overfit the data. However, a key aim of this study was to develop an analytic approach to this complex dataset that will be used to analyse data from the main study. The results from each of the four models are reasonably consistent and suggest insights into the association between housing condition and the number of skin infections that will be further explored with a larger dataset.

The extent to which the composite variables for the functional state of facilities for healthy living practices reflect the practical reality in households is likely to be limited. This is most evident in the rules used to define facilities required to conduct HLPs for washing children and removal of faeces for different age groups (Table 1). Furthermore, the difficulties of assessment of household composition in remote Indigenous communities in Australia is well documented [40-43], and the data collected in this study can at best be a partial reflection of a complex reality.

The analysis of skin health data is clearly only possible where adequate clinic records are available. The credibility of results from these analyses can also be undermined by systematic bias in the recording of health information for children of different social circumstances. The potential for such bias to influence study findings would be less in a follow-up study where children act as their own controls when analysing the change in infection rate before and after a housing intervention.

The absence of effect of the HLP variables reflecting facilities to wash children and to wash clothes warrants a closer examination of their construction and measurement. While the absence of these variables from any of the multivariate models may indicate that these factors are relatively weak determinants of skin infections, it is also possible that our definition and measurement of these variables has been inadequate. The inconsistency of the association of skin infection with contamination of the household environment is also expected to be at least partly a result of the difficulties of assessing contamination in an unobtrusive and sensitive way. The expected importance of contamination (internal and external) as an explanatory factor requires the development of accurate and appropriate approaches to measurement in order to refine the general explanatory power of this type of research. In addition, from a practical intervention perspective, the likely potential for reducing risk by minimizing contamination requires the development of appropriate skills in talking about this issue (i.e. hygiene) in a remote community context and research is one channel through which this development may be achieved.

In addition to refining the definition and measurement of variables included in this pilot study, the identification and inclusion of other important factors may enhance the potential value of future studies. For example, the apparent risk posed by older dwellings warrants closer examination through definition and measurement of variables that may explain this risk. Possible explanations include the type and quality of materials used in construction, the general condition of the structure (and the potential of structural materials to harbour infective materials); and features of housing design that may have changed over the years [32,44]. The information that may be obtained through the measurement and analysis of such variables may be particularly important to informing approaches to renovation of older dwellings as opposed to the construction of new dwellings. Similarly, identification and measurement of other important factors related to household composition and social processes may contribute to the understanding of how interventions to address these factors may enhance the health gains that may be achieved through infrastructure projects.

The study findings should be treated as appropriate to an exploratory pilot study. Confirmation of these findings in larger studies across a larger number of communities will be useful. Nevertheless, the findings do provide some guidance in the development of public health and preventive programs that aim to reduce the occurrence of skin infections in remote Indigenous communities. Key messages are that the provision of new and modern housing appears to be contributing to a reduction in skin infections, particularly where the housing programs lead to a reduction in crowding and the effective removal of human waste (i.e. having a functioning toilet). However, the capacity of infrastructure projects to improve health is likely to be limited in the absence of interventions that effectively address social and economic conditions. This conclusion is generally consistent both with an ecological understanding of the determinants of child health and with international experience [35]. The risk of skin infection associated with crowding of young children and their carers, with younger and older carers, low family income and with high child mobility is important in the development of criteria for housing allocation, social and health support programs and clinical awareness.

## Conclusion

The methods used in this pilot study were generally feasible, and the analytic approach provides for a meaningful interpretation that is consistent with contemporary international understandings of the impact of the social and physical environment on child health. Refinement of methods from the experience of this pilot study is expected to provide for a deeper level of understanding from a current larger follow-up study of the impact of housing improvements on child health. In general terms, the key areas for refinement of methods were: 1) development of a more comprehensive conceptual model that includes influences such as psycho-social measures for carers and householders, community status of the householder and measures of the condition of the wider community environment; and 2) development of more structured survey tools that incorporated a range of questions from widely used standard survey tools.

## Competing interests

Two of the authors (RB, MS) have conducted housing related evaluations under contract to the Northern Territory (NT) government. SG is an employee of the NT government. Financial support for this project was provided by the Indigenous Housing Authority of the NT.

## Authors' contributions

RB was responsible for the overall conception design, project management and drafting of the paper. MS made a major contribution to the development of the analytic approach, conducted the analyses, and contributed to the drafting of the paper. EM made a major contribution to project design, conducting the fieldwork, and contributed to the statistical analysis and drafting of the paper. SH contributed to the development of the analytic approach and to conducting the analysis. DB, GR and SG contributed to the conceptualisation and design of the study, and interpretation of the study findings. All authors read and approved the final manuscript.

## Pre-publication history

The pre-publication history for this paper can be accessed here:


